# Supplementary material for: Effects of Trichoderma harzianum combined with Phanerochaete chrysosporium on lignin degradation and humification during chicken manure and rice husk composting
Source: Front Microbiol. 2025 Feb 28;16:1515931. doi: 10.3389/fmicb.2025.1515931 (PMC11906335; doi:10.3389/fmicb.2025.1515931)
Supplement: Supplementary file 1 [file Data_Sheet_1.zip › 6.Function/All/FunGuild/l1/PCA_with_name.pdf]

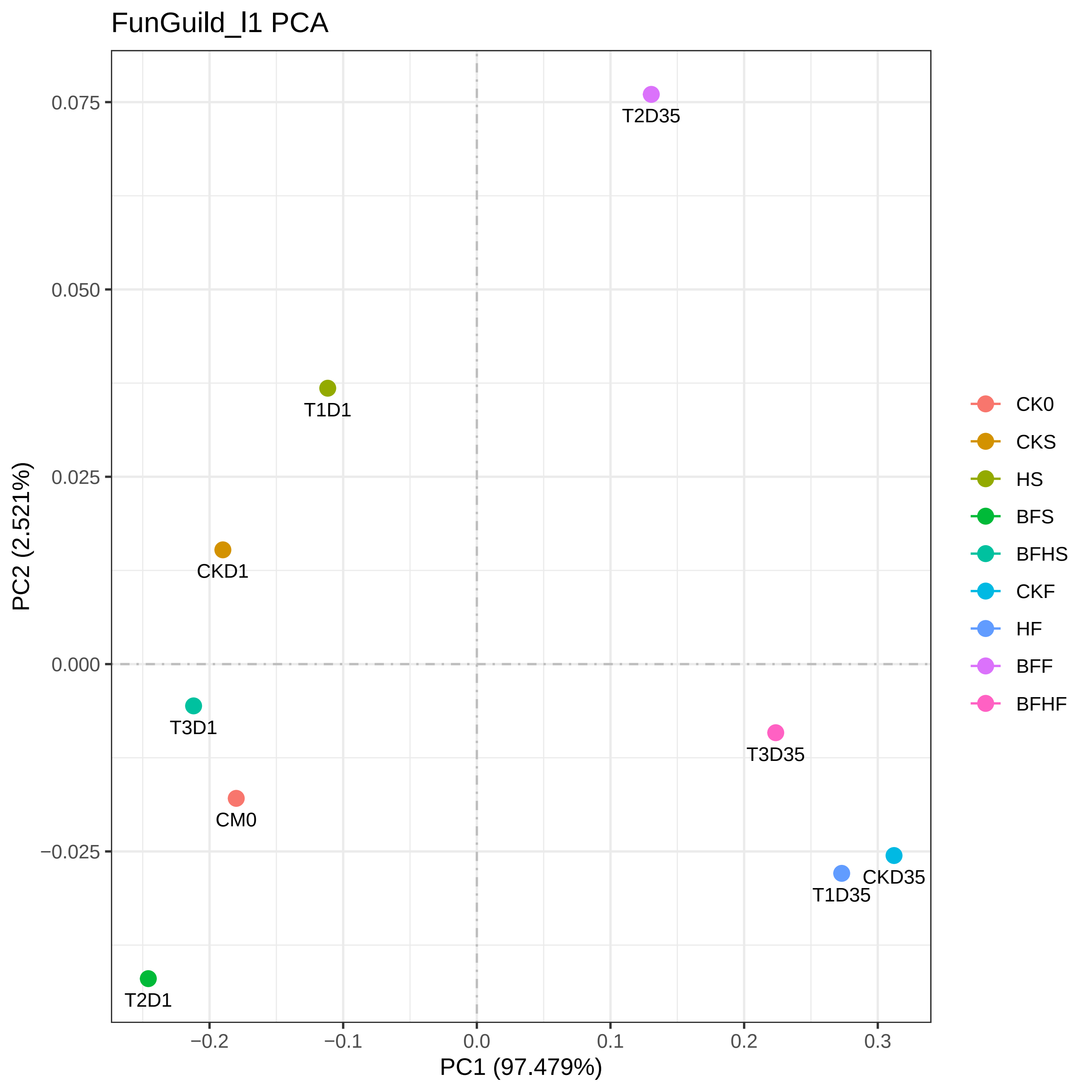
FunGuild_l1 PCA
PC1 (97.479%)
PC2 (2.521%)
0.075
0.050
0.025
0.000
−0.025
−0.2
−0.1
0.0
0.1
0.2
0.3
T2D35
T1D1
CKD1
T3D1
CM0
T2D1
T3D35
CKD35
T1D35
CK0
CKS
HS
BFS
BFHS
CKF
HF
BFF
BFHF
